# G Protein-Coupled Receptor 87 (GPR87) Promotes Cell Proliferation in Human Bladder Cancer Cells

**DOI:** 10.3390/ijms161024319

**Published:** 2015-10-14

**Authors:** Xia Zhang, Dage Liu, Yushi Hayashida, Homare Okazoe, Takeshi Hashimoto, Nobufumi Ueda, Mikio Sugimoto, Yoshiyuki Kakehi

**Affiliations:** 1 Department of Urology, Kagawa University Faculty of Medicine, 1750-1 Ikenobe, Miki-cho, Kita-gun, Kagawa 761-0793, Japan; E-Mails: rinda@med.kagawa-u.ac.jp (Y.H.); okazoe@me.com (H.O.); nob@med.kagawa-u.ac.jp (N.U.); micsugi@med.kagawa-u.ac.jp (M.S.); 2 Department of General Thoracic Surgery, Kagawa University Faculty of Medicine, 1750-1 Ikenobe, Miki-cho, Kita-gun, Kagawa 761-0793, Japan; E-Mail: dgliu@med.kagawa-u.ac.jp; 3 Department of Cardiovascular Physiology, Kagawa University Faculty of Medicine, 1750-1 Ikenobe, Miki-cho, Kita-gun, Kagawa 761-0793, Japan; E-Mail: hasimoty@med.kagawa-u.ac.jp

**Keywords:** GPR87, shRNA, Adenovirus, gene therapy, p53, xenograft

## Abstract

G protein-coupled receptor 87 (GPR87) is a newly deorphanized member of the cell surface molecule G protein-coupled receptor family. GPR signaling was shown to play a role in promotion of cell growth and survival, metastasis, and drug resistance. The overexpression of GPR87 has also been reported in many malignant tumors including bladder cancer. The aim of the present study is to examine the effect of silencing GPR87 expression with a replication-deficient recombinant adenoviral vector expressing short hairpin RNA targeting GPR87 (Ad-shGPR87) and to explore the underlying molecular mechanisms in bladder cancer cells. Six GPR87-expressing human bladder cancer cells, HT1197, HT1376, J82, RT112, TCCSUP and UMUC3, were used. Infection with Ad-shGPR87 effectively downregulated the GPR87 expression, and significantly reduced the percentage of viable cells in 4 of 6 cell lines as detected by an MTT assay. Significant inhibition on cell proliferation with Ad-shGPR87 was observed in the wild-type p53 bladder cancer cell lines (HT1197, RT112, TCCSUP and UMUC3), but not in the mutant p53 cells (HT1376 and J82). As represented by a wild-type p53 RT112 cell, Ad-shGPR87 infection significantly enhanced p53 and p21 expression and caused caspase-dependent apoptosis. Furthermore, the treatment with Ad-shGPR87 exerted a significant antitumor effect against the GPR87-expressing RT112 xenografts. GPR87 appeared to be a promising target for gene therapy, and Ad-shGPR87 had strong antitumor effects, specifically anti-proliferative and pro-apoptotic effects, against GPR87-expressing human bladder cancer cells.

## 1. Introduction

Bladder cancer is the most common malignancy of the urinary tract and the 9th leading course of malignancy in the world [1]. Most of bladder cancer occurs in males, and the highest incidence rates are found in the countries of Europe, North America, and Northern Africa and are variated up to 14-fold internationally [2]. More than 90% of bladder cancer is transitional cell carcinoma (TCC), or more properly, urothelial cell carcinoma [3]. Up to 80% of bladder cancers appear as non-muscular invasive tumors which frequently recur, and 5%–10% of them progress to muscle-invasive cancers. Despite the multidisciplinary treatment including surgery, chemotherapy and radiation, the five-year survival rate of patients with muscle-invasive bladder cancer is still about 50% [4]. Therefore, it is necessary to develop a novel approaches to conquer this disease.

G protein-coupled receptors (GPCRs) stucturely contain an extracellular NH_2_ terminus followed by a seven-membrane-spanning helix with three intracellular loops, and finally an intracellular COOH terminus [5]. It has been shown that GPCR signaling plays a role in promotion of cell growth and survival, metastasis, and drug resistance [6]. GPCRs are prime targets for therapeutic intervention in cancers, accounting for 50% of all drug targets [5]. Among these GPCRs, GPR87, a novel orphan receptor, was recently deorphanized and shown to be a lysophosphatidic acid receptor [7,8,9]. Use of various mRNA expression databases, laser-capture-microdissected (LCM) tissue samples and immunohistochemistry with a panel of human tumors have demonstrated tumor-specific overexpression of GPR87 in many human cancers, including TCC [10]. Moreover, our recent clinical study of non-muscle-invasive bladder cancer also showed that GPR87-overexpression was correlated with higher tumor proliferation, and that patients with GPR87-positive tumors had a shorter intravesical recurrence-free survival period [11]. The precise molecular mechanisms by which GPR87 promotes cell proliferation and contributes to cell viability have not been sufficiently analyzed. Glatt *et al.* [10] reported that GPR87 contributes to the viability of human tumor cells, and Zhang *et al.* [12] reported that GPR87-mediated signal transfection is necessary for p53-dependent cell survival in response to genotoxic stress.

To explore effective gene therapies for GPR87-expressing cancers including urothelial cancer, and to clarify the functional role of GPR87, we constructed an adenoviral vector expressing short hairpin RNA (shRNA) targeting GPR87 (Ad-shGPR87) and confirmed its anti-proliferative effect on an GPR87-expressing bladder cell line HT1197 [11]. In the present study, we further explored the antitumor activity in more bladder tumor cell lines both *in vitro* and *in vivo* and aim to clarify the functional role of GPR87 in bladder cancer. We found that this vector effectively inhibited the proliferation of GPR87-expressing cell lines both *in vitro* and *in vivo*. With this effective tool, we then analyzed the intracellular pathways to uncover the mechanism by which GPR87 is able to regulate the proliferation and survival of human bladder cancer cells. Consequently, knockdown of GPR87 led to a p53-dependent signal transduction and caused apoptosis in the bladder cancer cells. GPR87 may be a very good candidate target when developing new treatment strategies for patients with bladder cancer.

## 2. Results

### 2.1. GPR87 Expression in Human Bladder Cancer Cell Lines

**Figure 1:**
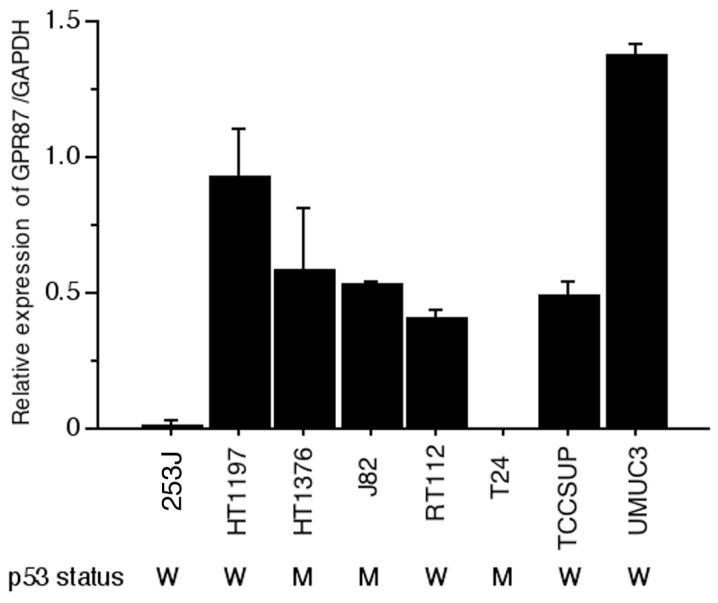
*GPR87* gene expression and p53 status in eight human bladder cancer cell lines. Relative expression levels of *GPR87/GAPDH* mRNA were assessed via real-time RT-PCR. W: wild-type; M: mutant type.

Although GPR87 was reported to be overexpressed in squamous cell carcinomas in different locations and in their lymph node metastases, including bladder cancer tissue [10], bladder cancer cell lines have not been adequately studied. The relative *GPR87* gene expression level referred to the internal control was evaluated in eight human bladder cancer cell lines. Six cell lines, HT1197, HT1376, J82, RT112, TCCSUP and UMUC3 cells, showed *GPR87* gene expression (6/8, 75.0%). However, two cell lines, 253J and T24 cells, showed very low levels of *GPR87* gene expression (2/8, 25.0%) (Figure 1). Most of human bladder cell lines showed GPR87 expression.

### 2.2. Ad-shGPR87 Efficiently Downregulates GPR87 Expression

**Figure 2:**
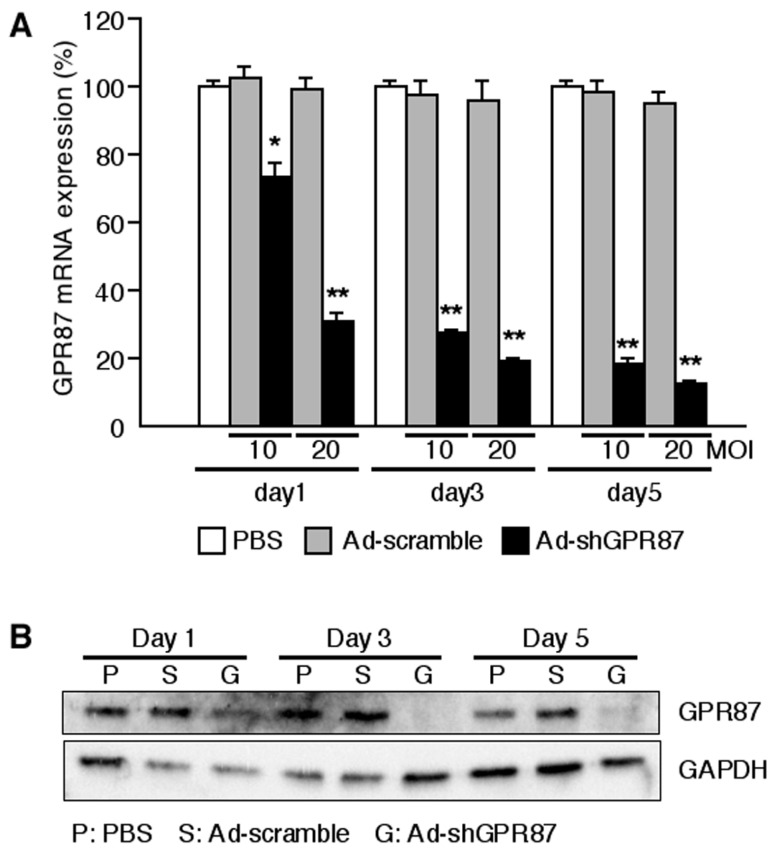
Expression of GPR87 in GPR87-expressing human bladder cancer cells RT112 after infection with adenoviral vectors. (**A**) Time-dependent and dose-dependent *GPR87* gene expressions in RT112 cell; (**B**) Time-dependent GPR87 protein expressions in RT112 cells. MOI: multiplicity of infection; * *p* < 0.05; ** *p* < 0.005, *vs.* Ad-scramble treatment.

**Figure 3:**
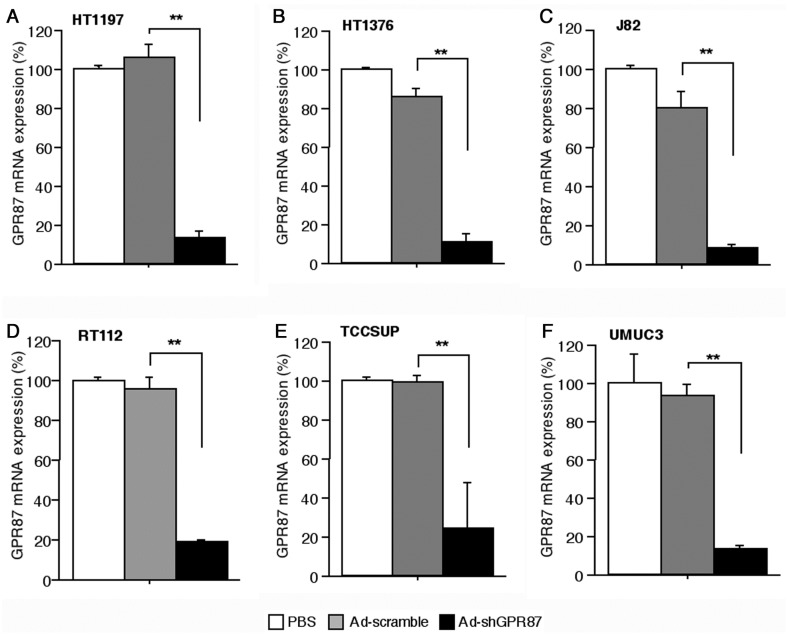
Ad-shGPR87 effectively knocked down *GPR87* gene expression in six GPR87-overexpressing human bladder cancer cell lines (HT1197 (**A**); HT1376 (**B**); J82 (**C**); RT112 (**D**); TCCSUP (**E**) and UMUC3 (**F**) cells). Gene expression of *GPR87* was assesed with real-time PCR 3 days after infection with Ad-shGPR87 at a MOI of 20. MOI: multiplicity of infection; ** *p* < 0.005, *vs.* Ad-scramble treatment.

As shown by RT112 cells, infection with Ad-shGPR87 at a multiplicity of infection (MOI, PFU/cell) of 10 and 20 effectively knocked down the *GPR87* gene expression in a time- and dose-dependent manner (*p* < 0.005 *vs.* Ad-scramble, respectively). The level of *GPR87* mRNA was strongly reduced from the first day after Ad-shGPR87 infection (Figure 2A). Downregulation of GPR87 protein was also detected after that of the *GPR87* gene decrease (Figure 2B). Ad-shGPR87 effectively knocked down *GPR87* gene expression in all of the six GPR87-overexpressing cancer cell lines (HT1197, HT1376, J82, RT112, TCCSUP and UMUC3 cells) 3 days after infection (Figure 3).

### 2.3. Ad-shGPR87 Inhibits Proliferation of GPR87-Expressing Cancer Cells

**Figure 4:**
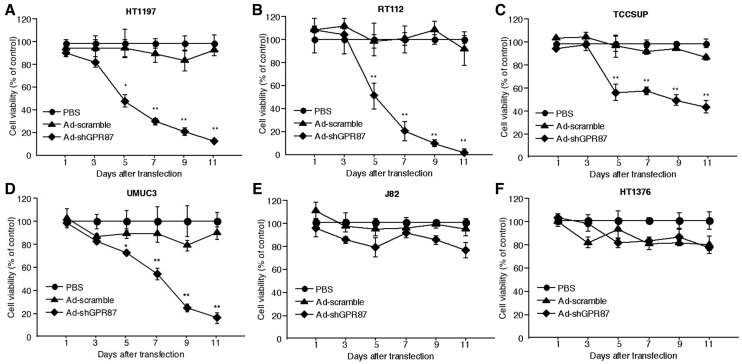
Cell viability evaluated by MTT assay in six GPR87-expressing bladder cancer cells. Ad-shGPR87 inhibited the proliferation of bladder cancer cells with the wild-type p53 (**A**–**D**), but not the cells with mutant p53 (**E**,**F**). Cells were infected with Ad-shGPR87 at a MOI of 20. Each cell viability assay was performed in triplicate and repeated three times. The cell viability of the treatment group was normalized with the value from the PBS treatment group and was shown as mean values ± SD. A comparison between Ad-shGPR87 and Ad-scramble group was carried out with the independent Student’s *t*-test on each individual day. MOI: multiplicity of infection; * *p* < 0.05; ** *p* < 0.005, *vs.* Ad-scramble treatment.

After gene expression analysis with real-time PCR, six GPR87-expressing cell lines were subjected to MTT analysis. Though Ad-shGPR87 significantly reduced the *GPR87* gene expression in all of these cell lines, the antiproliferative effect of Ad-shGPR87 varied (Figure 4). After Ad-shGPR87 infection, the percentages of viable cells significantly decreased in 4 of 6 cells, but was not obvious in 2 cell lines. These results indicate the essential role of GPR87 in regulating cell proliferation of bladder cancer cells. In referring to the p53 status, we found that only bladder cancer cells with wild type p53 showed significant inhibition in proliferation, not the cells with mutant type p53. This result indicates that p53 plays an essential role in GPR87 signal transduction.

### 2.4. Ad-shGPR87 Induces Apoptosis in GPR87-Overexpressing RT112 Cells

**Figure 5:**
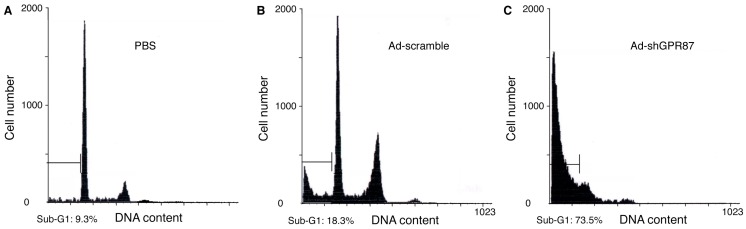
Flow cytometric analysis of propidium iodide-staining in GPR87-overexpressing RT112 cells 5 days after infection with Ad-shGPR87 at a MOI of 20. Apoptotic cells were represented by the fraction of cells in the sub-G1 phase. The apoptotic cells in the Ad-shGPR87-treated group (**C**) were significantly increased compared to the PBS-treated (**A**) and Ad-scramble-treated group (**B**). One of three experiments with similar results is shown.

**Figure 6:**
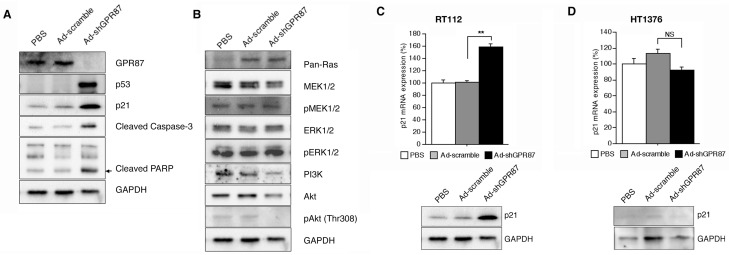
GPR87 promotes cell proliferation and inhibits apoptosis via the p53 pathway in RT112 cells. The RT112 cells were collected and analyzed 5 days after infection with Ad-shGPR87 at a MOI of 20. (**A**) The p53 and its downstream signal p21 protein expression severely increased. On the other hand; (**B**) the MAPK pathway was not changed, but the PI3K/Akt pathway was decreased significantly. The gene expression (**upper** panel) and protein expression (**lower** panel) of p53 downstream gene *p21* were also increased significantly in the wild-type p53 RT112 cells (**C**), but not in the mutant p53 HT1376 cells (**D**). Three independent experiments were performed for evaluating gene expression with real-time PCR or protein expression with western blot analyses. MOI: multiplicity of infection; ** *p* < 0.005, *vs.* Ad-scramble treatment; NS, not significant.

A flow cytometric analysis was performed using RT112 cells at 5 days after infection with Ad-shGPR87. The apoptotic cells were 9.3% and 18.3% in PBS-treated and Ad-scramble-treated control cells, and were 73.5% in the Ad-shGPR87-treated cells (Figure 5). In addition, western blot results also showed a higher level of cleaved caspase-3 and cleavaged-PARP protein in the Ad-shGPR87-treated cells as compared to the PBS-treated and Ad-scramble-treated RT112 cells (Figure 6A).

### 2.5. Ad-shGPR87 Exhibits an Antiproliferative Effect by Inhibiting Cell Proliferation and Inducing Apoptosis through the p53 Pathway

To explore the underlying mechanism of GPR87 in cell proliferation and survival, the p53-related signal pathway was investigated. After efficiently knocking down GPR87 with Ad-shGPR87 in the wild-type p53 RT112 cells, p53 protein expression was markedly enhanced. The enhancement of p53 protein expression was associated with an enhancement in p21 protein expression, caspase 3 and DNA damage-induced PARP cleavage (Figure 6A). To clarify how the GPR87 regulates the expression of tumor suppressor p53, we investigated the Rho GTPases, MAPK, and phosphatidylinositol 3-kinase (PI3K)/Akt pathway, by which GPRs activate a plethora of downstream signaling pathway. In comparison to the Rho GTPase and MAPK pathway, the PI3K/Akt pathway showed a remarkable change after inhibition of GPR87 with Ad-shGPR87 in RT112 cells. After efficiently knockdown by Ad-shGPR87, the PI3K and Akt protein significantly decreased in the Ad-shGPR87-treated cells as compared to those in PBS and Ad-scramble-treated cells (Figure 6B). Regarding the p53 status, the p53 downstream gene expression of p21 was increased by 1.5-fold in the wild-type p53 cells of RT112, but not in the mutant-type p53 HT1376 cell after Ad-shGPR87 infection (Figure 6C). Thus, p53 appears to be an essential downstream factor in GPR87 signal transduction.

### 2.6. Antitumor Activity of Ad-shGPR87 against GPR87-Expressing Tumor Xenografts

**Figure 7:**
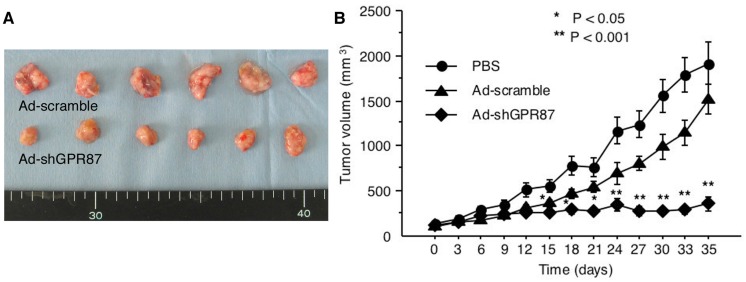
(**A**) Tumor appearances and (**B**) volumes of GPR87-expressing RT112 xenografts in scid mice for 35 days after Ad-shGPR87 treatment. The volumes of treatment group were shown as mean values ± SD and comparison between Ad-shGPR87 and Ad-scramble group was carried out with the independent Student’s *t*-test on each individual day. * *p* < 0.05; ** *p* < 0.005, *vs.* Ad-scramble treatment.

A GPR87-expressing RT112 tumor xenograft model was prepared in scid mice. The tumor volumes at day 35 were 1917.5 ± 633.0 mm^3^ in the PBS-treated groups, 1518.2 ± 442.5 mm^3^ in the Ad-scramble-treated groups and 361.8 ± 214.5 mm^3^ in the Ad-shGPR87 treated group. The Ad-shGPR87 treatment significantly inhibited the growth of RT112 xenografts in comparison to PBS treatment and Ad-scramble treatment (*p* < 0.005, *vs.* Ad-scramble, Figure 7).

## 3. Discussion

Human GPR87, also known as GPR95, was first identified by Wittenberger *et al.* [13] as an orphan recptor in 2001. Human GPR87 protein contains 358 amino acids encoded by hGPR87 gene locates on chromosome 3q24. Suggested potential ligands for GPR87 have included UDP-glucose and cysteinyl-leukotrienes and, most recently, LPA [7]. GPR87 is expressed at low levels in human tissues with the exception of placenta, and head and neck. Up-regulation of GPR87 was reported in squamous cell carcinomas (SCC) of the lung, cervix, skin, urinary bladder, testis, and head and neck [10,14].

Although emerging *in vitro* and *in vivo* data suggests that GPRs play important roles in the regulation of cell morphology, polarity and migration, few studies have investigated the function of GPR87. However, it has been suggested that GPR87 contributes to the viability of human cancer cells [10] and is necessary for p53-dependent cell survival in response to genotoxic stress [12]. A recent study by Yan *et al.* [15] also demonstrated that GPR87 promoted the growth and metastasis of CD133+ cancer stem-like cells in hepatocellular carcinoma.

Here we demonstrated both *in vitro* and *in vivo* that GPR87 contributes to the viability of human bladder cancer cells. The adenoviral vector expressing shRNA for silencing the expression of GPR87 effectively inhibited cell proliferation and induced apoptosis in GPR87-expressing bladder cancer cells. Our findings are consistent with those of Gatt *et al.* [10], who showed that silencing of GPR87 expression with small interfering RNA (siRNA) resulted in loss of cell viability in the cervical cancer cell HN5. Furthermore, our *in vivo* study demonstrated strong antitumor activity of Ad-shGPR87 against GPR87-overexpressing tumor xenografts for the first time. Our present results and those of others lend strong support to the possibility that GPR87 plays an important role in cancer cell survival and inhibits apoptosis, and that GPR87 expression is essential for the development and maintenance of cancer cells.

Few studies have investigated the regulation of GPR87 expression. Eight commercially available human bladder cancer cell lines were investigated. Six cell lines showed GPR87 gene expression and two cell lines, 253J and T24 cells, showed very low levels of GPR87 gene expression. The reason for the low or absent GPR87 expression in 253J and T24 cells is still unclear. In a recent study by Zhang *et al.* [12,16], GPR87 expression was reported to be regulated by the tumor suppressor p53 and by p53-dependent DNA damage in the cancer cell lines MCF7 and ROC. However, in the present study, we found that suppression of GPR87 in bladder cancer cell lines using Ad-shGPR87 triggered an increase in p53 expression in the absence of any stress due to anti-cancer drugs, followed by loss of cell viability through inhibition of cell proliferation and induction of apoptosis. The inibition of proliferation with Ad-shGPR87 can only be observed in the wild-type p53 bladder cell lines (HT1197, RT112 TCCSUP and UMUC3), and not in the mutant p53 cells (HT1376 and J82) (Figure 4). It appears that p53 is not a regulator for GPR87 expression, but rather a downstream effector of GPR87. After efficient knockdown by Ad-shGPR87, the expression of p53 protein was markedly enhanced, leading to significantly enhanced expression of the downstream targets of p53, p21 at both the gene and protein levels. Furthermore, our investigations of Rho GTPases, MAPK, and the phosphatidylinositol 3-kinase (PI3K)/Akt pathway, by which GPRs activate a variety of downstream signaling pathways [6], have shown that the PI3K/Akt pathway is markedly inhibited by knockdown of GPR87, in comparison with Rho GTPases and the MAPK pathway. As activation of the PI3K/Akt survival pathway has been reported to prevent p53 activation and p53-dependent apoptosis, [17,18] loss of GPR87 may trigger a decrease of Akt expression, which stabilizes the E3 ubiquitin ligase Mdm2, thereby inducing an increase in the level of p53 via the PI3K/Akt survival pathway. Further studies are necessary to clarify the pathways involved in GPR87-mediated survival.

Although RNAi is a valuable research tool and also a promising way to treat cancer, the successful application of RNAi to cancer gene therapy depends on the efficient delivery of siRNA into the cells [19,20,21]. An adenoviral vector expressing shRNA showed a stable expression of short interfering RNAs in mammalian cells [22,23]. Furthermore, adenoviral vectors have been widely used for the expression of transgenes, not only under experimental conditions [24,25] but also in a clinical setting [26]. Therefore, we constructed an adenoviral vector expressing shRNA targeting GPR87 to establish new treatment for tumors overexpressing GPR87. We demonstrated that this vector exerted effective activity against GPR87-expressing cells both *in vitro* and *in vivo*.

## 4. Experimental Section

### 4.1. Cell Lines

Eight human bladder cancer cell lines were investigated. HT1197, HT1376 J82, RT112, T24 and UMUC3 were obtained from ATCC^®^ through the Japanese official distributor. RT4 and TCCSUP were kindly given by Prof. Margaret Knowles, University of Leeds, UK. The p53 status of cells was summarized in Figure 1 according to the result of sequencing analysis and genotyping from our related laboratory, Kyoto University [27] and a constantly updated database named UMD p53 mutation database [28]. These cell lines were cultured in RPMI 1640 medium (SIGMA-ALDRICH, St. Louis, MO, USA) supplemented with 10% fetal bovine serum and 1% penicillin-streptomycin at 37 °C in a 5% CO_2_ atmosphere.

### 4.2. Construction of Adenoviral Vectors

An adenoviral vector expressing shRNA expressed under the control of the RNA polymerase III-dependent promoter for supplying stable siRNA molecules was constructed as described previously [11,29]. A control adenoviral vector expressing shRNA against the scramble sequence of GPR87-siRNA (5′-UCUUAAUCGCGUAUAAGGCTT-3′) was also constructed (Ad-scramble). Constructed adenoviral vectors were amplified in 293 HEKcells and purified by CsCl ultracentrifugation.

### 4.3. RNA Preparation and Real-Time RT-PCR

mRNA expression was determined by quantitative real-time PCR (qPCR) as previously described [11]. Briefly, total RNA from cells was isolated by TRizol RNA isolation reagents (Life Technologies, Carisbad, CA, USA) and used for cDNA synthesize using TaqMan reverse transcriptase kit (Applied Biosystems, Branchburg, NJ, USA). The qPCR was performed with the StepOnePlus Real-Time PCR System (Applied Biosystems, Foster City, CA, USA). The primers and probes were purchased from the Assays-on Demand Gene Expression Assay (GPR87 assay ID Hs00225057_m1, p21 ID assay Hs00355782_m1, Mdm2 assay ID Hs00234753_m1, Applied Biosystems). GAPDH (ID 4326317E, Applied Biosystems) was used as a internal control for normalization among cell samples.

### 4.4. Cell Proliferation

The *in vitro* cell viability test was performed with a 3-(4,5-demerthylthiazol-2-yl)-2, 5-diphenyltetrazolium bromide (MTT) assay as described previously [29]. Briefly, cells were seeded at a density of 4 × 10^3^ cells per well in 96-well culture plates. After 24 h, the medium was removed and the cells were infected at a multiplicity of infection (MOI) of 20 for 1 h. The cell viability was determined by MTT using a Cell Proliferation Kit I (Roche, Mannheim, Germany) at different time points. The cell viability in each well was measured in terms of optical density at a wavelength of 570 nm, with 750 nm as the reference wavelength. Each cell viability assay was performed in triplicate.

### 4.5. Western Blot Analysis

Western blotting was performed as described previously [29]. Cells were harvested and prepared in the Cell Lysis Buffer M (Wako Pure Chemical Industries, Ltd., Osaka, Japan), according to the manufacturer’s instructions. For detecting GPR87, a lysis buffer specially for extracting cellular membranes’ incorporate proteins (Cell-LyEX MP, Toyo Ink Group, Japan) was used. Protein concentration was measured by Bio-Rad Protein Assay Kit (Bio-Rad, Laboratories, Inc., Hercules, CA, USA). Protein samples (40 μg) were run on a 10% Mini-PROTAN TGXPrecast Gel (Bio-Rad) and transferred onto nitrocellulose membranes. After being blocked (Superblock T20, Thermo, Rockford, lL, USA) for 1 h. The membranes were incubated overnight with the primary specific antibodies detecting GPR87 (ab77517, Abcam, CamBridge, UK, 1:2000), p53 (DO-7, Daco, Aalborg, Denmark, 1:2000), p21 Waf1/Cip1 (12D1, CST, 1:1000), cleaved caspase 3 (Asp 175, CST, 1:1000), and cleaved PARP (Asp 214, CST, 1:1000). Antibodies for detecting Pan-Ras (F132, sc-32, 1:2500) and p-Akt1 (Thr 308, sc-135650, 1:500) were purchased from Santa Cruz Biotech. Antibodies for detecting MAPK pathway were all purchased from CST (MEK1/2, pMEK1/2, ERK1/2 and pERK1/2, all diluted at 1:1000). Antibody for detecting PI3K and Akt was purchased from a cell survival antibody Sample Kit (5004-1, Epitomics, Burlingame, CA, USA, 1:1000). A monoclonal antibody against GAPDH (G9545, Sigma, Tokyo, Japan, 1:2500) was used to control sample loading. The membranes were then incubated with HRP-labeled secondary antibodies for 1 h. The signals were developed in ECL Chemiluminescence Kit (Amersham Biosciences, Little Chalfont, Buckinghamshire, UK).

### 4.6. Flow Cytometric Analysis

The tested cells were collected and washed twice with PBS. Cells were fixed in 70% ethanol at 4 °C overnight. Fixed samples were treated with 250 mg/mL RNase A at 37 °C for 1 h and suspended in 60 μg/mL propidium iodide (PI) and incubated in the dark at room temperature for 30 min. The stained cells were analyzed with Cytomics FC500 (Beck-man coulter, Tokyo, Japan). Apoptotic cells were represented by the fraction of cells in the sub-G1 phase.

### 4.7. Xenograph Tumor Model in Scid Mice

The *in vivo* experiment was performed as described previously [29]. Briefly, tumor xenografts were prepared by implanting tumors derived from RT112 cells subcutaneously into the back of 6-week-old male scid mice (cr-Scid/Scid Jcl, Yokohama, Japan). When the tumor volume reached approximately 100 mm^3^, the mice were randomly divided into three groups (six mice/group): Ad-shGPR87, Ad-scramble, and a control group treated with PBS. Intratumoral injection with adenoviral vectors (at 2 × 10^9^ PFU, respectively) or 0.5 mL of PBS was performed every 4 days. Tumor growth was monitored every 4 days for 30 days by measuring tumor size with a caliper. The tumor volume was calculated by the following formula: tumor volume = (length) × (width)^2^ × 0.5. Animal experiments were performed in accordance with the Guide for the Care and Use of Laboratory Animals from Kagawa University.

### 4.8. Statistical Analysis

Data were presented as mean ± SD. The independent Student’s *t*-test or ANOVA was used to compare the continuous variables between the groups. Values of *p* < 0.05 were considered statistically significant.

## 5. Conclusions

In sum, knockdown of GPR87 led to a p53-dependent signal transduction and caused apoptosis in the bladder cancer cells. GPR87 may be a very good candidate target when developing new treatment strategies for patients with bladder cancer.

## Abbreviations

GPR87, G protein-coupled receptor 87; GPCR, G protein-coupled receptor; RT-PCR, reverse-transcription polymerase chain reaction; MTT, 3-(4,5-dimethylthiazol-2-yl)-2,5-diphenyltetrazolium bromide; shRNA, short hairpin RNA; cDNA, complementary DNA; GAPDH, glyceraldehyde 3-phosphate dehydrogenase; MOI, multiplicity of infection.
